# *Anti*-Addition Mechanism in the Intramolecular Hydroalkoxylation of Alkenes Catalyzed by PVP-Stabilized Nanogold

**DOI:** 10.3390/molecules17032579

**Published:** 2012-03-02

**Authors:** Hiroaki Kitahara, Hidehiro Sakurai

**Affiliations:** Research Center for Molecular Scale Nanoscience, Institute for Molecular Science, Myodaiji, Okazaki 444-8787, Japan; Email: ammuruga@ims.ac.jp

**Keywords:** gold nanoclusters, hydroalkoxylation, *anti*-addition, *π*-activation

## Abstract

(1R*,4S*,4aR*,9aS*,10S*)-10-Hydroxy-10-phenyl-1,4a,9a,10-tetrahydro-1,4-methanoanthracen-9(4*H*)-one (**1c**) was prepared for the elucidation of the reaction mechanism of intramolecular hydroalkoxylation of alkenes catalyzed by gold nanoclusters stabilized by a hydrophilic polymer, poly(*N*-vinyl-2-pyrrolidone) (**Au:PVP**). It was found that the reaction proceeded *via anti*-addition of alcohol to the alkene assisted by *π*-activation of the gold clusters, which is the same mechanism as the hydroamination by toluenesulfonamides.

## 1. Introduction

**Scheme 1:**
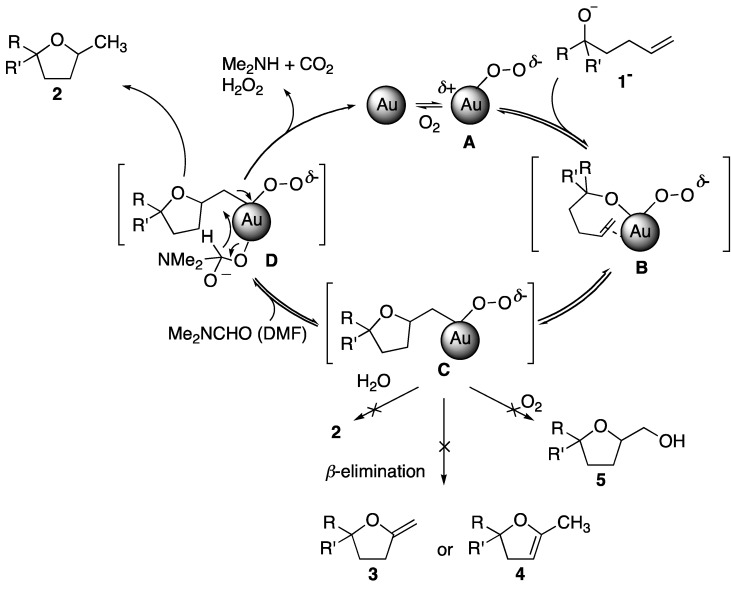
Possible mechanism of **Au:PVP**-catalyzed hydroalkoxylation of γ-hydroxyalkenes (**1**).

Since the discovery of Haruta’s CO oxidation reaction [1], aerobic oxidation reactions have been a central issue in nanogold chemistry in contrast to the chemistry of cationic gold(I) complexes, where the focus has been on their behavior as soft Lewis acid catalysts [2,3,4,5,6]. On the contrary, we have demonstrated that *quasi*-homogeneous nanogold protected by hydrophilic polymer, poly(*N*-vinyl-2-pyrrolidone) (**Au:PVP**), exhibits a formal “Lewis acid” activity and promotes intramolecular heterocyclization of γ-hydroxyalkenes and γ-aminoalkenes [7,8,9,10,11,12]. The success of the reaction depends on the choice of the sacrificial reductant (solvent) because the redox reaction between O_2_ and the solvent takes place behind the main (Lewis acidic) addition reaction. For example, DMF is found to be a good co-solvent with H_2_O in the reaction of hydroalkoxylation but EtOH or EtOH-H_2_O are more suitable in the case of hydroamination. Scheme 1 shows the proposed mechanism of the **Au:PVP**-catalyzed hydroalkoxylation. The reaction is initiated by the formation of key intermediate **A**, which possesses an electron-deficient site generated by adsorption of O_2_ onto the surface of the Au. Species **A** acts as a Lewis acid, activating both alkoxide and alkene by adsorption onto the surface (**B**), and giving **C** by either the insertion of an alkene into O-Au bond or the external attack of oxygen to the alkene adsorbed on Au. From this C-Au intermediate, neither β-elimination (Wacker-type process: **3** or **4**), O_2_ insertion (oxygenation: **5**), nor protonation proceeds; only DMF acts as a hydrogen source to afforded **2** accompanied by the regeneration of free Au clusters.

**Scheme 2:**
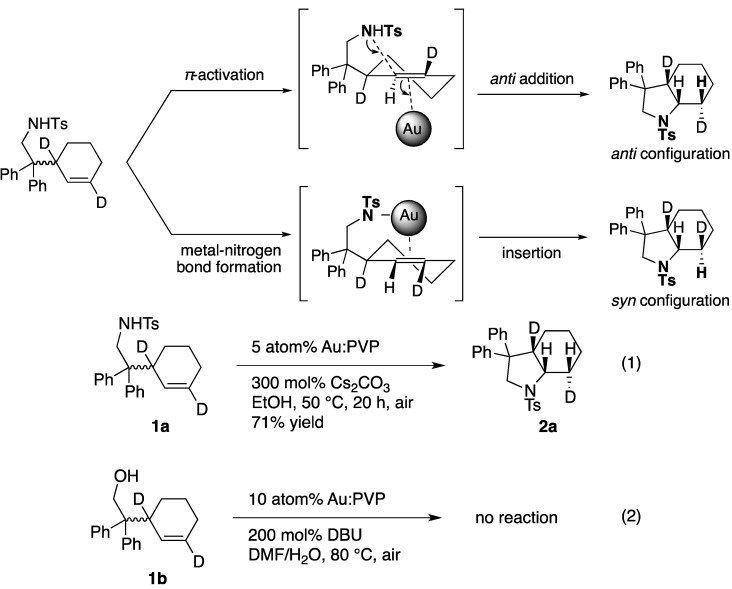
Two possible route to intramolecular hydroamination.

Among these intramolecular heterocyclizations, the reaction of toluenesulfonamides is well studied due to their high reactivity including the mechanism at the cyclization process [8,11]. Two main pathways are possible in the cyclization process, which may occur together: amine activation and alkene activation [13,14,15,16]. In one pathway, a metal amide is formed via amine activation by N-H oxidative addition, which then allows insertion of the alkene into the M-N bond (Scheme 2). These pathways have been proposed for organolanthanide [17], group IV [18,19,20,21], or Cu(II) complex [22] catalyst systems. An alternative mechanism involves olefin activation *via* a transition metal complex similar to cationic Au(Ι) complexes [23,24,25,26]. Finally it was elucidated that the reaction proceeds *via anti*-addition of the toluenesulfonamide followed by *π*-activation even under basic and protic solution conditions (Equation 1) [8,11].

To be honest, the result was different from our hypothesis. One reason is that the reaction was carried out under the basic conditions. In general, the *anti*-addition mechanism has been observed under (Lewis) acidic conditions [23,24]. Another is that adsorption of nitrogen or oxygen, equal to gold-nitrogen or gold-oxygen bond formation, is believed to be essential for this reaction by the analogy of the mechanism of alcohol oxidation. We have elucidated that the co-adsorption process of molecular oxygen and the alkoxide, generated by the deprotonation by the base, is facile and promotes the aerobic alcohol oxidation on the surface of Au_20_^-^ clusters, a model for **Au:PVP** [27,28,29]. However, the result of Equation (1) suggested that the adsorbed alkoxide (Au-O) does not attack to the alkenes but the external (non-adsorbed) alcohol would attack to the alkenes adsorbed on the Au surface if the same reaction mechanism is adapted to the hydroalkoxylation. Such confusion motivated us to investigate the same mechanistic study in the case of hydroalkoxylation.

## 2. Results and Discussion

**Scheme 3:**
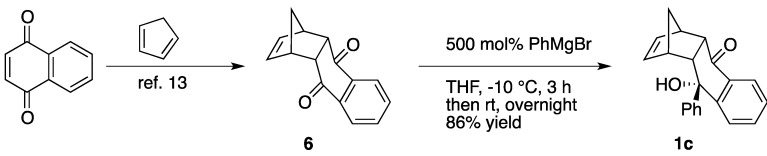
Preparation of γ-hydroxyalkene **1c**.

However, upon investigation a severe problem becomes evident. It has been reported that the hydroalkoxylation reactivity is highly susceptible to steric effects, especially at the substituents on alkene in comparison with hydroamination [9]. Indeed, the reaction of the alcohol **1b** did not give the cyclized product but rather a complex mixture including alcohol oxidation products (Equation 2). Therefore a new model compound, (1R*,4S*,4aR*,9aS*,10S*)-10-hydroxy-10-phenyl-1,4a,9a,10-tetrahydro-1,4-methanoanthracen-9(4*H*)-one (**1c**), was designed based on the following two features. One is that **1c** possesses a norbornene moiety, which might be highly reactive towards the addition reaction [13,14,15]. Also, we chose a α,α-diaryl tertiary alcohol like **1c** according to our previous results [9]. The precursor of **1c** (compound **6**) was prepared by the Diels-Alder reaction of 1,4-naphthoquinone with cyclopentadiene according to the literature [30]. Phenyl Grignard reagent attacked one of the carbonyl groups selectively from the *exo* side to afford the *endo* tertially alcohol in 86% yield (Scheme 3).

**Scheme 4:**
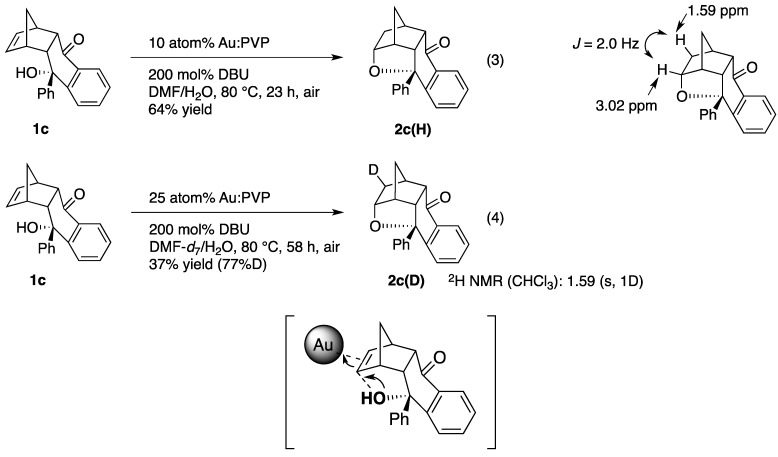
*Anti*-Addition mechanism in the **Au:PVP**-catalyzed hydroalkoxylation.

The first concern was the poor reactivity of **1c**. Indeed, no reaction was observed under the previously reported conditions (10 atom% **Au:PVP**, 200 mol% DBU, DMF/H_2_O, 50 °C). The cycloaddition took place when the temperature was increased to 80 °C, and **2c(H)** was obtained in 64% yield after 23 h (Scheme 4, Equation 3). A second concern was that a very large kinetic isotope effect has been observed in many case of the **Au:PVP**-catalyzed reactions [7,8,9,10,11,12,31]. Thus, the reaction proceeded very slowly when the solvent was replaced to DMF-*d*_7_/H_2_O in order to confirm the stereoselectivity. Finally the amount of **Au:PVP** was increased to 25 atom% to afford **2c(D)** in 37% yield after 58 h at 80°C (Scheme 4, Equation 4).

Judging from the mass spectrum, only one deuterium was introduced in this reaction with 77%D yield, similar to the previous result [8,9]. ^2^H-NMR showed a singlet peak at 1.59 ppm assignable to the *exo* position. Introduction of the D at the *exo* position was further confirmed by the coupling with adjacent *exo* proton (α position of the tetrahydrofuran ring). The proton in **2c(H)** was observed at 3.02 ppm as ddd (*J* = 2.0, 4.2, 10.4 Hz), but the smallest coupling (*J* = 2.0 Hz) corresponding to the vicinal *exo-exo* coupling disappeared in **2c(D)**. Although it is difficult to exclude the formation of the trace amount of the *endo*-D isomer under these experimental conditions, it can be concluded that *exo*-D should be the major product, which indicates that the hydroalkoxylation is also followed by the *anti*-addition mechanism as same as the reaction with toluenesulfonamide (Scheme 4).

Finally, some comments on the role of the base in this reaction are warranted because basic conditions are indispensable for the hydroalkoxylation. The present results reveal that adsorption of **1** through the deprotonated alkoxide is not required for promoting the reaction. In other words, the base should play an important role in a different process. The most probable step might be the hydrogen transfer process from the formyl group of DMF (**D** in Scheme 1). Under the basic conditions, DMF is easily adsorbed on the surface of gold via its hemiacetal form, from which a β-hydrogen might be released. The similar effect has also been proposed in the formylation of amine catalyzed by **Au:PVP** under basic conditions [32].

## 3. Experimental Section

Melting points were determined on a Yanaco HK-10D and were uncorrected. Infrared (IR) spectra were recorded on a JASCO FT IR-4100 spectrometer. ^1^H- and ^13^C-NMR spectra were measured on a JEOL JMN LAMBDA 400 spectrometer at 23 °C at 400 and 100 MHz, respectively. CDCl_3_ was used as a solvent and the residual solvent peaks were used as an internal standard (^1^H-NMR: 7.26 ppm; ^13^C-NMR: 77.00 ppm). ^2^H-NMR spectra were measured on JEOL JNM LAMBDA 500 spectrometer (500 MHz) in CHCl_3_. Silica gel chromatography was performed on Kanto 60N, Wako Wakosil C-300, or Yamazen Hi-Flash column using a Yamazen YFLC purification system. TLC analysis was performed using Merck Silica gel 60 F_254_ and preparative TLC was conducted using Wako Wakogel B-5F. **Au:PVP** was prepared according to the literature [34]. Other reagents and solvents were commercially purchased and further purified according to the standard methods, if necessary.

### 3.1. Preparation of *(1*R*,*4*S*,*4a*R*,*9a*S*,*10*S*)-10-Hydroxy-10-phenyl-1,4a,9a,10-tetrahydro-1,4-methanoanthracen-9(4H*)-one (**1c**)*

To a solution of **6** (224 mg, 1 mmol) in THF (25 mL) at −10 °C under argon atmosphere was slowly added 1 M PhMgBr (5 mL, 5 mmol) and the mixture was stirred at −10 °C for 3 h then at room temperature overnight. The reaction was quenched with H_2_O and the aqueous phase was extracted with EtOAc (3 × 20 mL) and then the combined organic layers were washed with H_2_O and brine, dried over Na_2_SO_4_, and concentrated *in vacuo*. The crude product was purified by silica gel chromatography (10–20% EtOAc/Hexane) to afford **1c** (259.3 mg, 86%). Pale yellow solid; mp 44–45 °C; IR (KBr) 3488, 2920, 1661 cm^−1^; ^1^H-NMR (CDCl_3_): *δ* 1.45–1.50 (m, 2H), 2.45 (s, 1H), 3.28–3.36 (m, 3H), 3.41 (s, 1H), 5.54 (dd, *J* = 2.4, 5.6 Hz, 1H), 5.76 (dd, *J* = 2.8, 5.6 Hz, 1H), 7.14–7.33 (m, 6H), 7.52–7.61 (m, 2H), 7.68–7.70 (m, 1H); ^13^C-NMR: *δ* 200.95, 149.56, 145.80, 134.85, 134.55, 134.50, 134.12, 128.58, 127.49, 127.27, 125.56, 125.24, 124.96, 75.03, 50.94, 50.85, 49.92, 49.82, 46.92; HRMS *m/z* Calcd for C_21_H_18_O_2_: 302.1307. Found: 302.1300.

### 3.2. Procedure for the Hydroalkoxylation

All the reactions were carried out using an EYELA PPS-2510 organic synthesizer. A test tube (*ϕ* = 30 mm) was placed with **1c** (0.1 mmol), DBU (30 μL, 200 mol%), and dried **Au:PVP** (86 mg = 10 atom%). Water (20 mL) and DMF (or DMF-*d*_7_) (10 mL) were added and the reaction mixture was stirred vigorously (1,300 rpm) at 80 °C for the time specified. The reaction mixture was extracted with ethyl acetate (3 × 20 mL), and then the combined organic layers were washed with water and brine, dried over Na_2_SO_4_, and concentrated *in vacuo*. Purification of the product **2c** was carried out by PTLC.

(2a^1^R*,4aS*,9bS*)-9b-phenyl-2a,2a^1^,3,4,4a,9b-hexahydro-2,4-methanobenzo[5,6]indeno[7,1-bc]furan-5(2H)-one [**2c(H)**]: Colorless solid; mp 160–161 °C; IR (KBr) 2921, 1678 1102, 1037 cm^−1^; ^1^H-NMR (CDCl_3_): *δ* 1.44–1.50 (m, 2H), 1.55–1.61 (m, 1H), 1.67-1.70 (m, 1H), 3.02 (ddd, *J* = 2.0, 4.2, 10.4 Hz, 1H), 3.13 (dd, *J* = 4.3, 10.4 Hz, 1H), 4.64 (dd, *J* = 6.1, 6.1 Hz, 1H), 6.71–6.76 (m, 1H), 7.09 (br, 1H), 7.32–7.41 (m, 5H), 7.82 (br, 1H), 8.03–8.07 (m, 1H); ^13^C-NMR: *δ* 199.54, 145.46, 144.74, 133.61, 133.17, 129.48, 128.29, 127.97, 127.19, 127.10, 126.78, 83.75, 80.33, 50.66, 49.18, 48.01, 39.39, 38.03, 36.99; HRMS *m/z* Calcd for C_21_H_18_O_2_: 302.1307. Found: 302.1310.

(2a^1^R*,4aS*,9bS*)-10-deuterio-9b-phenyl-2a,2a^1^,3,4,4a,9b-hexahydro-2,4-methanobenzo[5,6]indeno[7,1-bc]furan-5(2H)-on*e* [**2c(D)**]: ^1^H-NMR (CDCl_3_): *δ* 1.44–1.47 (m, 2H), 1.67–1.70 (m, 1H), 2.81–2.84 (m, 1H), 2.92 (d, J = 3.4 Hz, 1H), 3.02 (dd, J = 4.3, 10.4 Hz, 1H), 3.13 (dd, J = 4.2, 10.4 Hz), 4.63 (d, J = 5.1 Hz, 1H), 6.71–6.75 (m, 1H), 7.09 (br, 1H), 7.32–7.42 (m, 5H), 7.81 (br, 1H), 8.03–8.06 (m, 1H); ^2^H-NMR (CHCl_3_): *δ* 1.59 (s, 1D); HRMS *m/z* Calcd for C_21_H_17_DO_2_: 302.1370. Found: 302.1365.

## 4. Conclusions

In conclusion, the hydroalkoxylation catalyzed by **Au:PVP** proceeds via an *anti*-addition mechanism as same as hydroamination from toluenesulfoaminde. As a result, the formal behavior of **Au:PVP** resembles that of the cationic Au(I) complex catalyst, well-known to be a soft π-Lewis acid, even though **Au:PVP** is negatively charged and performs under basic conditions [33]. Therefore the **Au:PVP** catalyst system may be expected to be useful in new reactions, similar to those of cationic Au(I) catalysts, but which are only accessible under basic conditions.
